# Supplementary material for: Modeling glioblastoma heterogeneity as a dynamic network of cell states
Source: Mol Syst Biol. 2021 Sep 16;17(9):e10105. doi: 10.15252/msb.202010105 (PMC8444284; doi:10.15252/msb.202010105)
Supplement: Supplementary file 6 — Source Data for Figure 5 [file MSB-17-e10105-s004.zip › Figure5A_sourcedata/GSEA_3017/nosigdb_stateB.GseaPreranked.1622795021725/RICHARDS_NOWAKOWSKI_SCIENCE_2017_U2_UPREG.html]

Details for gene set RICHARDS\_NOWAKOWSKI\_SCIENCE\_2017\_U2\_UPREG[GSEA]

|  || Dataset | state43017 |
| Phenotype | NoPhenotypeAvailable |
| Upregulated in class | na\_pos |
| GeneSet | RICHARDS\_NOWAKOWSKI\_SCIENCE\_2017\_U2\_UPREG |
| Enrichment Score (ES) | 0.5011034 |
| Normalized Enrichment Score (NES) | 3.2458315 |
| Nominal p-value | 0.0 |
| FDR q-value | 0.0 |
| FWER p-Value | 0.0 |
Table: GSEA Results Summary

  

Fig 1: Enrichment plot: RICHARDS\_NOWAKOWSKI\_SCIENCE\_2017\_U2\_UPREG      
 Profile of the Running ES Score & Positions of GeneSet Members on the Rank Ordered List

  

| PROBE | GENE SYMBOL | GENE\_TITLE | RANK IN GENE LIST | RANK METRIC SCORE | RUNNING ES | CORE ENRICHMENT || 1 | HIST1H4C |  |  | 0 | 1.373 | 0.0085 | Yes |
| 2 | HIST1H1B |  |  | 1 | 1.272 | 0.0164 | Yes |
| 3 | TOP2A |  |  | 3 | 1.079 | 0.0205 | Yes |
| 4 | HIST1H1C |  |  | 4 | 1.041 | 0.0270 | Yes |
| 5 | HIST1H1E |  |  | 5 | 0.983 | 0.0331 | Yes |
| 6 | RRM2 |  |  | 7 | 0.875 | 0.0359 | Yes |
| 7 | SERPINE1 |  |  | 8 | 0.873 | 0.0413 | Yes |
| 8 | FAM111A |  |  | 10 | 0.813 | 0.0438 | Yes |
| 9 | UBE2C |  |  | 11 | 0.802 | 0.0487 | Yes |
| 10 | TPX2 |  |  | 12 | 0.801 | 0.0537 | Yes |
| 11 | NUSAP1 |  |  | 13 | 0.801 | 0.0587 | Yes |
| 12 | HMGB2 |  |  | 14 | 0.786 | 0.0636 | Yes |
| 13 | FBXO5 |  |  | 15 | 0.785 | 0.0684 | Yes |
| 14 | NCAPG |  |  | 16 | 0.778 | 0.0733 | Yes |
| 15 | SPC25 |  |  | 17 | 0.763 | 0.0780 | Yes |
| 16 | PRC1 |  |  | 18 | 0.756 | 0.0827 | Yes |
| 17 | CDCA2 |  |  | 19 | 0.751 | 0.0874 | Yes |
| 18 | ESCO2 |  |  | 20 | 0.742 | 0.0920 | Yes |
| 19 | TACC3 |  |  | 21 | 0.741 | 0.0966 | Yes |
| 20 | GTSE1 |  |  | 22 | 0.732 | 0.1011 | Yes |
| 21 | HJURP |  |  | 23 | 0.731 | 0.1056 | Yes |
| 22 | KIF23 |  |  | 24 | 0.721 | 0.1101 | Yes |
| 23 | LRRN3 |  |  | 25 | 0.704 | 0.1145 | Yes |
| 24 | ASPM |  |  | 26 | 0.695 | 0.1188 | Yes |
| 25 | NDC80 |  |  | 27 | 0.695 | 0.1231 | Yes |
| 26 | ATAD2 |  |  | 28 | 0.685 | 0.1274 | Yes |
| 27 | KIF15 |  |  | 29 | 0.684 | 0.1316 | Yes |
| 28 | ARHGAP11A |  |  | 30 | 0.683 | 0.1359 | Yes |
| 29 | KPNA2 |  |  | 33 | 0.676 | 0.1348 | Yes |
| 30 | CENPF |  |  | 34 | 0.670 | 0.1390 | Yes |
| 31 | KIF4A |  |  | 37 | 0.662 | 0.1379 | Yes |
| 32 | ANLN |  |  | 39 | 0.659 | 0.1394 | Yes |
| 33 | RAD51AP1 |  |  | 40 | 0.657 | 0.1434 | Yes |
| 34 | SMC4 |  |  | 41 | 0.655 | 0.1475 | Yes |
| 35 | MELK |  |  | 42 | 0.655 | 0.1516 | Yes |
| 36 | CENPE |  |  | 43 | 0.654 | 0.1556 | Yes |
| 37 | NUF2 |  |  | 44 | 0.651 | 0.1597 | Yes |
| 38 | PRR11 |  |  | 45 | 0.649 | 0.1637 | Yes |
| 39 | DLGAP5 |  |  | 46 | 0.647 | 0.1677 | Yes |
| 40 | KIFC1 |  |  | 47 | 0.645 | 0.1717 | Yes |
| 41 | KIF18A |  |  | 48 | 0.639 | 0.1757 | Yes |
| 42 | DHFR |  |  | 49 | 0.638 | 0.1797 | Yes |
| 43 | NCAPG2 |  |  | 50 | 0.636 | 0.1836 | Yes |
| 44 | CENPA |  |  | 51 | 0.636 | 0.1876 | Yes |
| 45 | CDCA5 |  |  | 52 | 0.632 | 0.1915 | Yes |
| 46 | CKAP2 |  |  | 53 | 0.628 | 0.1954 | Yes |
| 47 | CCNA2 |  |  | 54 | 0.626 | 0.1993 | Yes |
| 48 | AURKA |  |  | 55 | 0.617 | 0.2031 | Yes |
| 49 | FANCI |  |  | 56 | 0.615 | 0.2069 | Yes |
| 50 | CKAP2L |  |  | 59 | 0.611 | 0.2055 | Yes |
| 51 | CENPU |  |  | 60 | 0.605 | 0.2092 | Yes |
| 52 | CENPK |  |  | 62 | 0.603 | 0.2104 | Yes |
| 53 | TYMS |  |  | 63 | 0.601 | 0.2141 | Yes |
| 54 | BUB1B |  |  | 64 | 0.596 | 0.2178 | Yes |
| 55 | CCDC34 |  |  | 65 | 0.595 | 0.2215 | Yes |
| 56 | KIF2C |  |  | 66 | 0.591 | 0.2252 | Yes |
| 57 | IGFBP5 |  |  | 67 | 0.590 | 0.2288 | Yes |
| 58 | SMC2 |  |  | 68 | 0.586 | 0.2325 | Yes |
| 59 | CDK1 |  |  | 69 | 0.585 | 0.2361 | Yes |
| 60 | PMP22 |  |  | 70 | 0.576 | 0.2397 | Yes |
| 61 | H2AFX |  |  | 71 | 0.576 | 0.2432 | Yes |
| 62 | CKS1B |  |  | 72 | 0.575 | 0.2468 | Yes |
| 63 | RACGAP1 |  |  | 75 | 0.572 | 0.2451 | Yes |
| 64 | FOXM1 |  |  | 76 | 0.570 | 0.2487 | Yes |
| 65 | MAD2L1 |  |  | 77 | 0.569 | 0.2522 | Yes |
| 66 | PLK1 |  |  | 78 | 0.567 | 0.2557 | Yes |
| 67 | DBF4 |  |  | 79 | 0.567 | 0.2592 | Yes |
| 68 | MMP2 |  |  | 80 | 0.565 | 0.2628 | Yes |
| 69 | CTGF |  |  | 81 | 0.564 | 0.2663 | Yes |
| 70 | CDCA8 |  |  | 82 | 0.562 | 0.2697 | Yes |
| 71 | CKS2 |  |  | 83 | 0.558 | 0.2732 | Yes |
| 72 | ECT2 |  |  | 84 | 0.558 | 0.2767 | Yes |
| 73 | KIF14 |  |  | 85 | 0.557 | 0.2801 | Yes |
| 74 | E2F7 |  |  | 86 | 0.556 | 0.2836 | Yes |
| 75 | AURKB |  |  | 89 | 0.548 | 0.2818 | Yes |
| 76 | SPC24 |  |  | 91 | 0.544 | 0.2825 | Yes |
| 77 | CENPM |  |  | 92 | 0.544 | 0.2859 | Yes |
| 78 | CCNB1 |  |  | 93 | 0.544 | 0.2893 | Yes |
| 79 | NCAPH |  |  | 94 | 0.543 | 0.2927 | Yes |
| 80 | PBK |  |  | 95 | 0.540 | 0.2960 | Yes |
| 81 | DEPDC1 |  |  | 97 | 0.536 | 0.2967 | Yes |
| 82 | XRCC2 |  |  | 98 | 0.530 | 0.3000 | Yes |
| 83 | SKA3 |  |  | 99 | 0.525 | 0.3033 | Yes |
| 84 | TMEM106C |  |  | 100 | 0.523 | 0.3065 | Yes |
| 85 | RRM1 |  |  | 103 | 0.520 | 0.3045 | Yes |
| 86 | MXD3 |  |  | 104 | 0.519 | 0.3078 | Yes |
| 87 | CMC2 |  |  | 105 | 0.516 | 0.3110 | Yes |
| 88 | CCNF |  |  | 106 | 0.513 | 0.3141 | Yes |
| 89 | SDC2 |  |  | 107 | 0.513 | 0.3173 | Yes |
| 90 | CDKN3 |  |  | 108 | 0.513 | 0.3205 | Yes |
| 91 | CEP135 |  |  | 109 | 0.513 | 0.3237 | Yes |
| 92 | UBE2T |  |  | 110 | 0.512 | 0.3269 | Yes |
| 93 | CDCA3 |  |  | 111 | 0.511 | 0.3300 | Yes |
| 94 | DIAPH3 |  |  | 112 | 0.510 | 0.3332 | Yes |
| 95 | BUB1 |  |  | 113 | 0.508 | 0.3364 | Yes |
| 96 | TUBB4B |  |  | 114 | 0.507 | 0.3395 | Yes |
| 97 | RAD21 |  |  | 115 | 0.506 | 0.3427 | Yes |
| 98 | BIRC5 |  |  | 116 | 0.504 | 0.3458 | Yes |
| 99 | CLSPN |  |  | 117 | 0.503 | 0.3489 | Yes |
| 100 | DTYMK |  |  | 118 | 0.503 | 0.3520 | Yes |
| 101 | CEP152 |  |  | 119 | 0.502 | 0.3551 | Yes |
| 102 | ASF1B |  |  | 120 | 0.501 | 0.3583 | Yes |
| 103 | PSMC3IP |  |  | 122 | 0.495 | 0.3587 | Yes |
| 104 | KIF18B |  |  | 123 | 0.494 | 0.3618 | Yes |
| 105 | DUT |  |  | 124 | 0.494 | 0.3648 | Yes |
| 106 | E2F8 |  |  | 125 | 0.493 | 0.3679 | Yes |
| 107 | SPDL1 |  |  | 126 | 0.492 | 0.3710 | Yes |
| 108 | TMPO |  |  | 127 | 0.492 | 0.3740 | Yes |
| 109 | ITGB3BP |  |  | 128 | 0.490 | 0.3771 | Yes |
| 110 | LMNB2 |  |  | 129 | 0.488 | 0.3801 | Yes |
| 111 | SKA1 |  |  | 131 | 0.487 | 0.3805 | Yes |
| 112 | SHCBP1 |  |  | 133 | 0.484 | 0.3809 | Yes |
| 113 | GMNN |  |  | 135 | 0.484 | 0.3813 | Yes |
| 114 | NCAPD2 |  |  | 136 | 0.483 | 0.3843 | Yes |
| 115 | TTK |  |  | 137 | 0.482 | 0.3873 | Yes |
| 116 | FANCB |  |  | 138 | 0.478 | 0.3903 | Yes |
| 117 | CENPL |  |  | 139 | 0.478 | 0.3932 | Yes |
| 118 | INCENP |  |  | 140 | 0.476 | 0.3962 | Yes |
| 119 | MDFIC |  |  | 141 | 0.476 | 0.3991 | Yes |
| 120 | C21orf58 |  |  | 142 | 0.470 | 0.4020 | Yes |
| 121 | CYR61 |  |  | 145 | 0.465 | 0.3997 | Yes |
| 122 | CD44 |  |  | 146 | 0.464 | 0.4026 | Yes |
| 123 | CDCA4 |  |  | 148 | 0.461 | 0.4028 | Yes |
| 124 | FANCD2 |  |  | 149 | 0.461 | 0.4057 | Yes |
| 125 | ORC6 |  |  | 150 | 0.461 | 0.4086 | Yes |
| 126 | KIF22 |  |  | 152 | 0.459 | 0.4088 | Yes |
| 127 | NCAPD3 |  |  | 153 | 0.458 | 0.4116 | Yes |
| 128 | CDC45 |  |  | 154 | 0.456 | 0.4145 | Yes |
| 129 | KNSTRN |  |  | 155 | 0.456 | 0.4173 | Yes |
| 130 | SPAG5 |  |  | 156 | 0.455 | 0.4201 | Yes |
| 131 | CCL2 |  |  | 157 | 0.455 | 0.4230 | Yes |
| 132 | MCM8 |  |  | 158 | 0.454 | 0.4258 | Yes |
| 133 | GPC6 |  |  | 159 | 0.454 | 0.4286 | Yes |
| 134 | ANXA1 |  |  | 160 | 0.453 | 0.4314 | Yes |
| 135 | CEP78 |  |  | 161 | 0.451 | 0.4342 | Yes |
| 136 | POLQ |  |  | 162 | 0.450 | 0.4370 | Yes |
| 137 | CDKN2C |  |  | 164 | 0.445 | 0.4372 | Yes |
| 138 | RPA1 |  |  | 165 | 0.445 | 0.4399 | Yes |
| 139 | TK1 |  |  | 167 | 0.444 | 0.4401 | Yes |
| 140 | BRIP1 |  |  | 168 | 0.443 | 0.4428 | Yes |
| 141 | BRCA1 |  |  | 171 | 0.440 | 0.4403 | Yes |
| 142 | BRCA2 |  |  | 174 | 0.436 | 0.4378 | Yes |
| 143 | SMCHD1 |  |  | 175 | 0.436 | 0.4405 | Yes |
| 144 | RBBP8 |  |  | 176 | 0.436 | 0.4432 | Yes |
| 145 | DEPDC1B |  |  | 177 | 0.436 | 0.4459 | Yes |
| 146 | MKI67 |  |  | 179 | 0.436 | 0.4460 | Yes |
| 147 | FEN1 |  |  | 180 | 0.435 | 0.4487 | Yes |
| 148 | ELN |  |  | 182 | 0.433 | 0.4488 | Yes |
| 149 | RHNO1 |  |  | 183 | 0.432 | 0.4515 | Yes |
| 150 | CDC20 |  |  | 184 | 0.431 | 0.4542 | Yes |
| 151 | USP1 |  |  | 185 | 0.430 | 0.4568 | Yes |
| 152 | MIS18BP1 |  |  | 186 | 0.430 | 0.4595 | Yes |
| 153 | DDIAS |  |  | 187 | 0.428 | 0.4622 | Yes |
| 154 | MGME1 |  |  | 188 | 0.428 | 0.4648 | Yes |
| 155 | BARD1 |  |  | 189 | 0.427 | 0.4675 | Yes |
| 156 | CDKN2D |  |  | 190 | 0.427 | 0.4701 | Yes |
| 157 | HMMR |  |  | 191 | 0.426 | 0.4728 | Yes |
| 158 | CKAP5 |  |  | 192 | 0.426 | 0.4754 | Yes |
| 159 | MND1 |  |  | 193 | 0.424 | 0.4780 | Yes |
| 160 | NEK2 |  |  | 194 | 0.423 | 0.4807 | Yes |
| 161 | NEDD1 |  |  | 195 | 0.423 | 0.4833 | Yes |
| 162 | MIS18A |  |  | 196 | 0.421 | 0.4859 | Yes |
| 163 | MCM4 |  |  | 199 | 0.418 | 0.4833 | Yes |
| 164 | DSN1 |  |  | 202 | 0.413 | 0.4806 | Yes |
| 165 | TCF19 |  |  | 207 | 0.410 | 0.4727 | Yes |
| 166 | ANXA2 |  |  | 211 | 0.408 | 0.4674 | Yes |
| 167 | ZWINT |  |  | 212 | 0.407 | 0.4699 | Yes |
| 168 | AXL |  |  | 213 | 0.407 | 0.4725 | Yes |
| 169 | LIG1 |  |  | 214 | 0.407 | 0.4750 | Yes |
| 170 | CCNE2 |  |  | 215 | 0.406 | 0.4775 | Yes |
| 171 | H2AFZ |  |  | 216 | 0.405 | 0.4800 | Yes |
| 172 | HSPB11 |  |  | 218 | 0.401 | 0.4799 | Yes |
| 173 | MNS1 |  |  | 219 | 0.401 | 0.4824 | Yes |
| 174 | UTRN |  |  | 220 | 0.400 | 0.4849 | Yes |
| 175 | CENPO |  |  | 223 | 0.398 | 0.4821 | Yes |
| 176 | TPM1 |  |  | 225 | 0.397 | 0.4820 | Yes |
| 177 | ITGB5 |  |  | 226 | 0.396 | 0.4844 | Yes |
| 178 | MORC4 |  |  | 229 | 0.395 | 0.4817 | Yes |
| 179 | STIL |  |  | 231 | 0.393 | 0.4815 | Yes |
| 180 | POC1A |  |  | 234 | 0.390 | 0.4787 | Yes |
| 181 | EMP1 |  |  | 235 | 0.390 | 0.4811 | Yes |
| 182 | VRK1 |  |  | 238 | 0.388 | 0.4783 | Yes |
| 183 | CDC6 |  |  | 239 | 0.388 | 0.4807 | Yes |
| 184 | PARPBP |  |  | 240 | 0.387 | 0.4831 | Yes |
| 185 | TRIP13 |  |  | 241 | 0.387 | 0.4855 | Yes |
| 186 | PCNA |  |  | 242 | 0.386 | 0.4879 | Yes |
| 187 | HMGN2 |  |  | 243 | 0.386 | 0.4903 | Yes |
| 188 | POLA1 |  |  | 247 | 0.383 | 0.4848 | Yes |
| 189 | RTKN2 |  |  | 248 | 0.379 | 0.4872 | Yes |
| 190 | CCNB2 |  |  | 250 | 0.379 | 0.4869 | Yes |
| 191 | POLD3 |  |  | 252 | 0.377 | 0.4867 | Yes |
| 192 | MPHOSPH9 |  |  | 254 | 0.375 | 0.4864 | Yes |
| 193 | PLK4 |  |  | 255 | 0.374 | 0.4887 | Yes |
| 194 | EPS8 |  |  | 257 | 0.371 | 0.4884 | Yes |
| 195 | C18orf54 |  |  | 259 | 0.371 | 0.4881 | Yes |
| 196 | RFC5 |  |  | 260 | 0.370 | 0.4904 | Yes |
| 197 | CENPJ |  |  | 261 | 0.370 | 0.4927 | Yes |
| 198 | GAS2L3 |  |  | 262 | 0.369 | 0.4950 | Yes |
| 199 | TUBB |  |  | 263 | 0.368 | 0.4973 | Yes |
| 200 | GINS4 |  |  | 265 | 0.367 | 0.4969 | Yes |
| 201 | CENPW |  |  | 267 | 0.366 | 0.4966 | Yes |
| 202 | TUBG1 |  |  | 268 | 0.365 | 0.4988 | Yes |
| 203 | CEP57L1 |  |  | 269 | 0.364 | 0.5011 | Yes |
| 204 | SMC1A |  |  | 274 | 0.361 | 0.4929 | No |
| 205 | CHAF1A |  |  | 276 | 0.359 | 0.4925 | No |
| 206 | MMS22L |  |  | 277 | 0.359 | 0.4948 | No |
| 207 | ZFP36L2 |  |  | 279 | 0.358 | 0.4944 | No |
| 208 | ANP32E |  |  | 280 | 0.358 | 0.4966 | No |
| 209 | GINS1 |  |  | 283 | 0.357 | 0.4936 | No |
| 210 | CDC25B |  |  | 286 | 0.356 | 0.4906 | No |
| 211 | CALM2 |  |  | 290 | 0.354 | 0.4849 | No |
| 212 | ZWILCH |  |  | 292 | 0.353 | 0.4845 | No |
| 213 | HAUS8 |  |  | 293 | 0.353 | 0.4867 | No |
| 214 | DCBLD2 |  |  | 294 | 0.351 | 0.4889 | No |
| 215 | RFC4 |  |  | 295 | 0.351 | 0.4911 | No |
| 216 | NEIL3 |  |  | 304 | 0.345 | 0.4723 | No |
| 217 | DTL |  |  | 305 | 0.345 | 0.4745 | No |
| 218 | PRKDC |  |  | 306 | 0.344 | 0.4766 | No |
| 219 | CDC25C |  |  | 307 | 0.343 | 0.4787 | No |
| 220 | CENPN |  |  | 309 | 0.343 | 0.4783 | No |
| 221 | FAM111B |  |  | 314 | 0.342 | 0.4699 | No |
| 222 | OIP5 |  |  | 315 | 0.341 | 0.4720 | No |
| 223 | DEK |  |  | 316 | 0.341 | 0.4742 | No |
| 224 | PRRX1 |  |  | 318 | 0.339 | 0.4737 | No |
| 225 | PHF19 |  |  | 319 | 0.339 | 0.4758 | No |
| 226 | AKAP12 |  |  | 320 | 0.339 | 0.4779 | No |
| 227 | CSE1L |  |  | 321 | 0.338 | 0.4800 | No |
| 228 | RFC3 |  |  | 322 | 0.338 | 0.4821 | No |
| 229 | PRDX6 |  |  | 323 | 0.338 | 0.4842 | No |
| 230 | HAUS6 |  |  | 324 | 0.337 | 0.4863 | No |
| 231 | CIT |  |  | 326 | 0.336 | 0.4857 | No |
| 232 | PRSS23 |  |  | 329 | 0.334 | 0.4826 | No |
| 233 | RFC2 |  |  | 331 | 0.333 | 0.4820 | No |
| 234 | EMP2 |  |  | 333 | 0.333 | 0.4815 | No |
| 235 | LMF2 |  |  | 338 | 0.330 | 0.4731 | No |
| 236 | UBR7 |  |  | 340 | 0.330 | 0.4725 | No |
| 237 | STAT1 |  |  | 341 | 0.328 | 0.4746 | No |
| 238 | PHGDH |  |  | 344 | 0.328 | 0.4714 | No |
| 239 | LRRCC1 |  |  | 349 | 0.326 | 0.4630 | No |
| 240 | SPARC |  |  | 350 | 0.325 | 0.4650 | No |
| 241 | HAUS3 |  |  | 351 | 0.325 | 0.4670 | No |
| 242 | HAT1 |  |  | 352 | 0.324 | 0.4690 | No |
| 243 | TMEM237 |  |  | 353 | 0.324 | 0.4710 | No |
| 244 | TRAM2 |  |  | 354 | 0.324 | 0.4730 | No |
| 245 | ARHGAP11B |  |  | 356 | 0.323 | 0.4724 | No |
| 246 | TACC1 |  |  | 359 | 0.322 | 0.4692 | No |
| 247 | FSTL1 |  |  | 362 | 0.321 | 0.4660 | No |
| 248 | XPO1 |  |  | 365 | 0.321 | 0.4627 | No |
| 249 | WDR34 |  |  | 366 | 0.321 | 0.4647 | No |
| 250 | RBBP7 |  |  | 367 | 0.321 | 0.4667 | No |
| 251 | UACA |  |  | 368 | 0.320 | 0.4687 | No |
| 252 | TOPBP1 |  |  | 369 | 0.319 | 0.4707 | No |
| 253 | MID1 |  |  | 375 | 0.317 | 0.4596 | No |
| 254 | ACTN4 |  |  | 376 | 0.316 | 0.4616 | No |
| 255 | NCAPH2 |  |  | 377 | 0.315 | 0.4635 | No |
| 256 | PIN1 |  |  | 379 | 0.314 | 0.4629 | No |
| 257 | ID3 |  |  | 389 | 0.311 | 0.4413 | No |
| 258 | ID1 |  |  | 390 | 0.311 | 0.4432 | No |
| 259 | KIF20A |  |  | 392 | 0.311 | 0.4426 | No |
| 260 | HAUS2 |  |  | 393 | 0.311 | 0.4445 | No |
| 261 | TMEM245 |  |  | 394 | 0.310 | 0.4464 | No |
| 262 | CPNE8 |  |  | 397 | 0.309 | 0.4431 | No |
| 263 | RHEB |  |  | 404 | 0.307 | 0.4293 | No |
| 264 | LRR1 |  |  | 410 | 0.304 | 0.4182 | No |
| 265 | LIN54 |  |  | 411 | 0.303 | 0.4201 | No |
| 266 | MPC2 |  |  | 412 | 0.303 | 0.4219 | No |
| 267 | PTTG1 |  |  | 413 | 0.302 | 0.4238 | No |
| 268 | ZGRF1 |  |  | 415 | 0.302 | 0.4231 | No |
| 269 | CD47 |  |  | 419 | 0.300 | 0.4171 | No |
| 270 | SMC5 |  |  | 420 | 0.300 | 0.4190 | No |
| 271 | CCNE1 |  |  | 424 | 0.299 | 0.4130 | No |
| 272 | AHI1 |  |  | 425 | 0.299 | 0.4148 | No |
| 273 | ZMYM1 |  |  | 429 | 0.298 | 0.4089 | No |
| 274 | NUP107 |  |  | 430 | 0.298 | 0.4107 | No |
| 275 | LAMC1 |  |  | 438 | 0.295 | 0.3943 | No |
| 276 | GINM1 |  |  | 439 | 0.295 | 0.3961 | No |
| 277 | MEST |  |  | 441 | 0.294 | 0.3953 | No |
| 278 | G2E3 |  |  | 446 | 0.294 | 0.3867 | No |
| 279 | IQGAP3 |  |  | 447 | 0.294 | 0.3885 | No |
| 280 | TEX30 |  |  | 448 | 0.294 | 0.3903 | No |
| 281 | CCDC150 |  |  | 449 | 0.293 | 0.3922 | No |
| 282 | MZT1 |  |  | 456 | 0.291 | 0.3783 | No |
| 283 | SYNE1 |  |  | 458 | 0.291 | 0.3775 | No |
| 284 | SEMA3A |  |  | 460 | 0.290 | 0.3767 | No |
| 285 | SAE1 |  |  | 463 | 0.289 | 0.3733 | No |
| 286 | RFWD3 |  |  | 464 | 0.289 | 0.3750 | No |
| 287 | ING2 |  |  | 466 | 0.289 | 0.3742 | No |
| 288 | NUP155 |  |  | 468 | 0.288 | 0.3734 | No |
| 289 | KCTD9 |  |  | 471 | 0.287 | 0.3700 | No |
| 290 | PLOD2 |  |  | 474 | 0.286 | 0.3665 | No |
| 291 | ACTN1 |  |  | 475 | 0.286 | 0.3683 | No |
| 292 | YEATS4 |  |  | 476 | 0.286 | 0.3701 | No |
| 293 | CHEK1 |  |  | 477 | 0.286 | 0.3718 | No |
| 294 | RNASEH2A |  |  | 478 | 0.285 | 0.3736 | No |
| 295 | NUCKS1 |  |  | 483 | 0.285 | 0.3649 | No |
| 296 | ARID5B |  |  | 484 | 0.285 | 0.3667 | No |
| 297 | HADH |  |  | 485 | 0.284 | 0.3685 | No |
| 298 | APOLD1 |  |  | 487 | 0.284 | 0.3676 | No |
| 299 | METTL4 |  |  | 488 | 0.283 | 0.3694 | No |
| 300 | POLH |  |  | 492 | 0.282 | 0.3633 | No |
| 301 | CTDSPL2 |  |  | 495 | 0.282 | 0.3598 | No |
| 302 | RBMS1 |  |  | 500 | 0.281 | 0.3511 | No |
| 303 | CENPH |  |  | 501 | 0.281 | 0.3529 | No |
| 304 | MYOF |  |  | 502 | 0.280 | 0.3546 | No |
| 305 | ZNF83 |  |  | 504 | 0.280 | 0.3537 | No |
| 306 | PSMC3 |  |  | 506 | 0.280 | 0.3529 | No |
| 307 | EFEMP2 |  |  | 507 | 0.280 | 0.3546 | No |
| 308 | IKBIP |  |  | 508 | 0.279 | 0.3563 | No |
| 309 | FAM83D |  |  | 511 | 0.279 | 0.3529 | No |
| 310 | IFITM3 |  |  | 512 | 0.279 | 0.3546 | No |
| 311 | WDHD1 |  |  | 513 | 0.278 | 0.3563 | No |
| 312 | TTF2 |  |  | 514 | 0.278 | 0.3580 | No |
| 313 | COL5A2 |  |  | 516 | 0.278 | 0.3572 | No |
| 314 | MBNL2 |  |  | 517 | 0.278 | 0.3589 | No |
| 315 | YBX3 |  |  | 519 | 0.278 | 0.3580 | No |
| 316 | CDC27 |  |  | 520 | 0.278 | 0.3597 | No |
| 317 | LSM3 |  |  | 522 | 0.277 | 0.3588 | No |
| 318 | DNMT1 |  |  | 523 | 0.277 | 0.3605 | No |
| 319 | CNN3 |  |  | 527 | 0.276 | 0.3544 | No |
| 320 | NFIC |  |  | 531 | 0.276 | 0.3483 | No |
| 321 | NUCB2 |  |  | 533 | 0.275 | 0.3474 | No |
| 322 | FIGNL1 |  |  | 542 | 0.273 | 0.3282 | No |
| 323 | SCLT1 |  |  | 548 | 0.273 | 0.3169 | No |
| 324 | CALU |  |  | 552 | 0.272 | 0.3107 | No |
| 325 | CNN2 |  |  | 556 | 0.271 | 0.3046 | No |
| 326 | ERCC6L |  |  | 557 | 0.271 | 0.3062 | No |
| 327 | DONSON |  |  | 559 | 0.271 | 0.3053 | No |
| 328 | BRD8 |  |  | 562 | 0.270 | 0.3018 | No |
| 329 | GGH |  |  | 564 | 0.270 | 0.3008 | No |
| 330 | CDK2 |  |  | 565 | 0.270 | 0.3025 | No |
| 331 | TMTC3 |  |  | 569 | 0.269 | 0.2963 | No |
| 332 | SAPCD2 |  |  | 578 | 0.268 | 0.2771 | No |
| 333 | CCDC18 |  |  | 586 | 0.267 | 0.2605 | No |
| 334 | SLC43A3 |  |  | 590 | 0.265 | 0.2543 | No |
| 335 | IFRD1 |  |  | 594 | 0.264 | 0.2481 | No |
| 336 | ARHGEF39 |  |  | 595 | 0.264 | 0.2498 | No |
| 337 | RAD1 |  |  | 607 | 0.262 | 0.2227 | No |
| 338 | KDELC2 |  |  | 612 | 0.261 | 0.2138 | No |
| 339 | OXCT1 |  |  | 614 | 0.261 | 0.2129 | No |
| 340 | HMGA2 |  |  | 619 | 0.260 | 0.2040 | No |
| 341 | SPATS2L |  |  | 620 | 0.260 | 0.2056 | No |
| 342 | NDE1 |  |  | 623 | 0.259 | 0.2020 | No |
| 343 | CRYBG3 |  |  | 625 | 0.259 | 0.2010 | No |
| 344 | PLSCR1 |  |  | 627 | 0.259 | 0.2000 | No |
| 345 | CHCHD3 |  |  | 628 | 0.259 | 0.2016 | No |
| 346 | CENPI |  |  | 630 | 0.258 | 0.2006 | No |
| 347 | ZNF85 |  |  | 635 | 0.258 | 0.1918 | No |
| 348 | MAGT1 |  |  | 642 | 0.257 | 0.1777 | No |
| 349 | CEP44 |  |  | 643 | 0.256 | 0.1793 | No |
| 350 | ASH2L |  |  | 644 | 0.256 | 0.1809 | No |
| 351 | CHN1 |  |  | 645 | 0.256 | 0.1825 | No |
| 352 | EXOSC8 |  |  | 647 | 0.256 | 0.1814 | No |
| 353 | UHRF1 |  |  | 652 | 0.255 | 0.1726 | No |
| 354 | DESI2 |  |  | 654 | 0.255 | 0.1716 | No |
| 355 | NUP37 |  |  | 655 | 0.255 | 0.1731 | No |
| 356 | FN3KRP |  |  | 656 | 0.255 | 0.1747 | No |
| 357 | RDX |  |  | 658 | 0.255 | 0.1737 | No |
| 358 | RPN1 |  |  | 662 | 0.254 | 0.1674 | No |
| 359 | MCC |  |  | 664 | 0.253 | 0.1664 | No |
| 360 | GPAA1 |  |  | 665 | 0.253 | 0.1680 | No |
| 361 | RASSF8 |  |  | 667 | 0.253 | 0.1669 | No |
| 362 | RAD51 |  |  | 670 | 0.252 | 0.1633 | No |
| 363 | USP13 |  |  | 673 | 0.252 | 0.1596 | No |
| 364 | FLNA |  |  | 676 | 0.251 | 0.1560 | No |
| 365 | MYBL1 |  |  | 678 | 0.251 | 0.1549 | No |
| 366 | SGCE |  |  | 680 | 0.250 | 0.1538 | No |
| 367 | VIM |  |  | 692 | -0.267 | 0.1268 | No |
| 368 | ITGB1 |  |  | 693 | -0.271 | 0.1285 | No |
| 369 | GDI2 |  |  | 697 | -0.274 | 0.1223 | No |
| 370 | FLRT3 |  |  | 699 | -0.275 | 0.1214 | No |
| 371 | RPL23 |  |  | 704 | -0.283 | 0.1127 | No |
| 372 | MMP16 |  |  | 707 | -0.285 | 0.1093 | No |
| 373 | ZEB1 |  |  | 717 | -0.317 | 0.0878 | No |
| 374 | TSPO |  |  | 723 | -0.345 | 0.0768 | No |
| 375 | HAS2 |  |  | 733 | -0.389 | 0.0558 | No |
| 376 | DDX21 |  |  | 745 | -0.440 | 0.0298 | No |
| 377 | PDGFRA |  |  | 758 | -0.673 | 0.0026 | No |
Table: GSEA details [plain text format]

  

Fig 2: RICHARDS\_NOWAKOWSKI\_SCIENCE\_2017\_U2\_UPREG: Random ES distribution      
 Gene set null distribution of ES for **RICHARDS\_NOWAKOWSKI\_SCIENCE\_2017\_U2\_UPREG**
